# Editorial: Sleep health: research and intervention perspectives

**DOI:** 10.3389/fpsyt.2023.1304094

**Published:** 2023-12-07

**Authors:** Matthew R. Cribbet, Ryuji Furihata, Masatsugu Sakata

**Affiliations:** ^1^Department of Psychology, College of Arts and Sciences, University of Alabama, Tuscaloosa, AL, United States; ^2^Agency for Student Support and Disability Resources, Kyoto University, Kyoto, Japan; ^3^Department of Health Promotion and Human Behavior, Kyoto University School of Public Health, Kyoto, Japan

**Keywords:** sleep, sleep health, intervention, risk factors, socioecological

Sleep health is a multidimensional construct that includes, but is not limited to, satisfaction or quality, sleepiness/alertness, timing, efficiency, duration, regularity and rhythmicity (1). These dimensions of sleep health can be adapted to individual, social and environmental demands for any individual at any point in time. A sleep health framework is important, as it can inform which specific sleep characteristics should be considered in mechanistic studies, and it could lead to the development of sleep treatments aimed at reducing morbidity and mortality.

We offer this Research Topic as an opportunity to highlight cutting-edge sleep health research, with an emphasis on risk factors for poor sleep health and ways to improve sleep. The seven papers published in this Research Topic include six empirical studies and one expert consensus paper on the treatment of Insomnia Disorder. The studies in this Research Topic exclusively focus on adult samples, but are diverse in age, race/ethnicity and health status. Examined together, the papers in this Research Topic can be placed along the translational spectrum (excluding basic science) and within a broader socioecological framework. Below we highlight some of the key findings and conceptual issues that arose from these papers.

## Socioecological risk factors for poor sleep health

The data from two papers in this Research Topic (Zeng et al.; Wang et al.) describe key socioecological risk factors impacting sleep quality. These papers address risk factors for poor sleep health during emerging adulthood, throughout midlife, and among individuals with varying health status. The papers highlight the idea that sleep health can be adapted to individual, social and environmental demands for any individual at any point in time. From a socioecological perspective, risk factors for poor sleep health can range from interpersonal and community factors to broader societal influences (2). As Wang et al. report, both bedtime habits and neighborhood environment were strongly associated with sleep quality in a sample of emerging adults. The effects of bedtime habits and neighborhood environment on poor sleep quality were attributed to low self-control and high stress. These findings are important, as they underscore important aspects of executive functioning central to stress regulation during a key developmental transition (3). In a large sample of Chinese men and women with inflammatory bowel disease, older age and depressed mood were associated with the presence and severity of poor sleep quality. If findings like these were expanded to include multiple sleep health dimensions, we might expect the number of sleep health dimensions to be associated in a gradient fashion with greater depressive symptoms (4).

Sleep health may also be impacted by key aspects of close relationships like marriage. In this Research Topic, Guo et al. found that compared to those who were unmarried, married men had shorter sleep duration, worse sleep quality, reported more daytime sleepiness, and endorsed more dysfunctional beliefs and attitudes about sleep. These findings are inconsistent with the literature on marriage and sleep (5) and with the broader marriage and health literature, which shows that men often benefit more than women across a variety of health outcomes (6). However, follow-up analyses revealed that those men who were married but living apart from a spouse due to military duties were driving the effects stated above. This is consistent with a small but growing literature demonstrating that separation from a married partner has detrimental impacts on sleep health [e.g., (7)].

In contrast, individual difference factors, such as personality, are also important for sleep health (8). Work in this Research Topic by Uygur et al. found associations between Type D personality, defined as the interaction of negative affectivity and social inhibition, and insomnia severity. These associations were partially mediated by sleep effort, poor sleep hygiene and sleep reactivity. When testing associations between personality and health outcomes it is important to address methodological concerns present in prior research on Type D personality traits with the goal of moving toward a more systematic, cumulative and practically applicable science of personality and health (9).

Finally, in a study that examined how aspects of sleep health might be associated with key health outcomes among individuals with intracranial tumors, perioperative sleep quality and sleep depth were associated with anxiety, depression, nausea and length of hospital stay post-surgery (Liu et al.). This study highlights the importance of sleep in clinical settings, especially for outcomes that directly impact recovery from medical procedures and the associated quality of life for patients.

## Interventions

Two papers in this Research Topic focused on how to improve sleep health. In a randomized controlled trail of a low-threshold sleep intervention consisting of three conditions (sleep data feedback plus sleep education, sleep data feedback alone, or no intervention) the authors (Eigl et al.) found small but beneficial effects of sleep health parameters. Moreover, these authors determined that personal contact may be a particularly important factor for promoting sleep health. Moving forward, it will be important to balance low cost, highly accessible interventions with ones that include more in-person contact with a sleep medicine provider. As we consider ways to address the sleep health needs of rural and underserved populations ways to maintain personal contact with care providers will be increasingly important. Finally, a paper on treatment strategies for Insomnia Disorder from Japanese expert consensus found that orexin receptor antagonists and sleep hygiene education were first-line treatment recommendations. While these recommendations are not based on scientific evidence and do not align with recommendations from the American Academy of Sleep Medicine (AASM) for treating Insomnia Disorder (10), they have clinical utility for sleep medicine practitioners working in Japan.

## Conclusion

Taken together these papers highlight the key factors that impact sleep health, offer treatment options to promote healthy sleep and reinforce the idea that sleep health can be adapted to individual, social and environmental needs for any individual at any point in time. Future considerations for research and practice include examining how multiple sleep health domains, when examined simultaneously, can influence health and wellbeing.

## Author contributions

MC: Conceptualization, Writing – original draft, Writing – review & editing. RF: Conceptualization, Writing – review & editing. MS: Conceptualization, Writing – review & editing.
